# Maternal mental health and child problem behaviours: disentangling the role of depression and borderline personality dysfunction

**DOI:** 10.1192/bjpo.bp.117.005843

**Published:** 2017-11-30

**Authors:** Fay Huntley, Nicola Wright, Andrew Pickles, Helen Sharp, Jonathan Hill

**Affiliations:** **Fay Huntley**, ClinPsyD, PhD, Department of Psychological Sciences, Institute of Psychology, Health and Society, , University of Liverpool, UK; **Nicola Wright**, BSc, MSc, Department of Psychological Sciences, Institute of Psychology, Health and Society, University of Liverpool, UK; **Andrew Pickles**, FMedSci, Professor, Biostatistics Department, Institute of Psychiatry, Psychology and Neuroscience, King’s College London, London, UK; **Helen Sharp**, DClinPsy, PhD, Department of Psychological Sciences, Institute of Psychology, Health and Society, University of Liverpool, Liverpool, UK; **Jonathan Hill**, FRCPsych, Professor, School of Psychology and Clinical Language Sciences, University of Reading, Reading, UK

## Abstract

**Background:**

It is not known whether associations between child problem behaviours and maternal depression can be accounted for by comorbid borderline personality disorder (BPD) dysfunction.

**Aim:**

To examine the contributions of maternal depression and BPD symptoms to child problem behaviours.

**Method:**

Depression trajectories over the first-year postpartum were generated using repeated measurement from a general population sample of 997 mothers recruited in pregnancy. In a stratified subsample of 251, maternal depression and BPD symptoms were examined as predictors of child problem behaviours at 2.5 years.

**Results:**

Child problem behaviours were predicted by a high maternal depression trajectory prior to the inclusion of BPD symptoms. This association was no longer significant after the introduction of BPD symptoms.

**Conclusions:**

Risks for child problem behaviours currently attributed to maternal depression may arise from more persistent and pervasive difficulties found in borderline personality dysfunction.

**Declaration of interest:**

None.

**Copyright and usage:**

© The Royal College of Psychiatrists 2017, this is an open access article distributed under the terms of the Creative Commons Attribution (CC BY) license.

Early-onset ‘life course persistent’ externalising child behaviours are associated with antisocial outcomes in adulthood and also with personality dysfunction and psychiatric disorders.^1,2^ They are therefore a major focus for early intervention. The association between maternal depression and child outcomes has received considerable research attention.^3–5^ Maternal depression during the child’s first year of life has been of particular interest because of concerns that exposure at this time may be particularly harmful through its influences on early mother–child interactions.^6–8^ Applications of longitudinal modelling techniques have enabled chronic exposures to depression, which may carry highest risk^9–11^ to be examined, and these have confirmed the link between maternal depression and child problem behaviours.^9,12^ Associations between maternal depression and child symptoms may, however, be confounded with other environmental and familial risks.^3,13^ A key question is whether associations could be better explained by mothers’ personality dysfunction, specifically borderline personality disorder (BPD) pathology, both because of its high comorbidity with depression^14,15^ and because it is characterised by interpersonal dysfunction and emotional regulation difficulties that may impair parenting.^16–18^ Consistent with this possibility, elevated externalising and internalising symptoms were associated with questionnaire-based self-reports of maternal borderline, antisocial and narcissistic symptoms in a cross-sectional study of 4-year-olds.^19^ No studies to date have examined prospectively the relative contributions of maternal depression over the postnatal period and BPD symptoms to young children’s problem behaviours.

If risks to children associated with BPD dysfunction were confined to mothers meeting diagnostic criteria, then even strong associations with problem behaviours would be of limited relevance to the general population. The dimensional approach to personality disorder is likely to be more relevant and generalisable to community mother–child dyad samples, where rates of diagnosable personality disorder are likely to be low but there may be substantial variation in subthreshold symptoms.^20^ Dimensional approaches to the personality disorders have been widely used,^21–23^ and in the case of BPD, subthreshold levels of symptoms have been associated with a range of impairments.^23–26^

The aim of this study was to examine whether elevated maternal depressive symptoms over the first year of life predict child externalising behaviours at 2.5 years, and whether this association is explained by symptoms of BPD. Internalising and total problem scores were also examined in light of recent evidence that findings apparently specific to externalising symptoms may reflect associations with a broader set of symptoms or with general psychopathology ‘p’.^27^

## Method

### Sample

Participants were members of the Wirral Child Health and Development Study, a prospective epidemiological longitudinal study starting in pregnancy. The study uses a two-stage stratified design in which a consecutive general population sample (the ‘extensive’ sample) is used to generate a smaller ‘intensive’ sample stratified by psychosocial risk and both are followed in tandem. The extensive sample was identified from consecutive first-time mothers who booked for antenatal care at the sole provider of universal prenatal care on the Wirral.

All women gave written informed consent at the point of recruitment in the antenatal clinic. Ethical approval for the study was granted by the Cheshire North and West Research Ethics Committee on 27 June 2006, reference number 05/Q1506/107. The cohort comprised 1233 mothers with surviving singleton babies. Mean age of the mothers at recruitment was 26.8 years (s.d.=5.8, range 18–51), 41.8% of the extensive sample were in the most deprived quintile of UK neighbourhoods^28^ and 96.1% were White British. Of these, 997 reported on depression symptoms on at least two postnatal assessment occasions and made up the sample for trajectory analyses. Maternal responses to questions about psychological abuse in their current or recent partner relationship^29^ were used to generate the stratified intensive sample of mothers for more detailed study. The sample stratification has been described in more detail previously.^30^ There were 316 mothers recruited to the stratified intensive sample at 32 weeks’ pregnancy. We focus here on the 251 mother–child dyads who completed the lab assessment when their children were 31.37 (s.d.=2.50) months old (‘2.5 years’). Mothers providing information at 2.5 years were slightly older (mean=27.9 years, s.d.=6.2, range 18–51) and less deprived (37.8% in most deprived quintile) than the original extensive sample.

### Measures

#### Maternal depression

Exposure to maternal depression was assessed by self-report using the Edinburgh Postnatal Depression Scale^31^ (EPDS) at 5, 9 and 29 weeks, and 14 months, and these scores were used to generate depression trajectories. The EPDS from age 2.5 years was also used in analyses to control for possible biasing of maternal reports of child problems.

#### Maternal BPD symptoms

Maternal BPD symptoms were assessed using the Structured Clinical Interview for DSM-IV Axis II Disorders^32^ (SCID-II). The SCID-II was administered when mothers were 32 weeks pregnant to assess symptoms of four personality disorders: borderline, antisocial, dependent and avoidant. Only BPD symptoms were examined in this study. Presence or absence of each symptom is initially assessed using a screening questionnaire (administered in this study at 20 weeks), and this is followed by a semi-structured interview to elicit further information regarding the extent each symptom has been persistent and pervasive over the previous 5 years, and whether it has caused functional impairment. Dimensional scores for maternal BPD were derived by summing the scores for each item: scored 1 (absent), 2 (subthreshold) and 3 (present).^33^ Scores ranged from 9 to 19. Ratings were made from audio recordings. The first author was trained to reliability in scoring the SCID-II and had experience of using it in a range of clinical and community samples. Interrater reliability based on 20 audio recordings from this study was high (intraclass correlation coefficient=0.91).

#### Child problem behaviours

Maternal report of child problem behaviours was assessed at 2.5 years using the preschool Child Behavior Checklist (CBCL), which has been extensively used in studies of child and adolescent emotional and behavioural disorders.^34^ It has 99 items, each scored 0 (not true), 1 (somewhat or sometimes true) and 2 (very true or often true), which are summed to create seven syndrome scales. Syndrome scales for externalising, internalising and total problem behaviours were used. Raw scores were used for analysis.

### Stratifier and potential confounders

#### Maternal negative emotionality

Maternal negative emotionality was assessed using the negative temperament scale from the Schedule for Nonadaptive and Adaptive Personality (SNAP). SNAP negative emotionality is strongly associated with measures of neuroticism.^35^

#### Child negative emotionality

Infant negative emotionality was assessed at 29 weeks and 14 months by maternal report using the distress to limitations and fear subscales of the Infant Behavioral Questionnaire – Revised (IBQ-R).^36^ The two subscales are combined and a mean score is used for analysis. The IBQ-R has established reliability and validity and has been widely used in developmental studies.^37,38^

#### Partner psychological abuse

Partner psychological abuse, used in the sample stratification, was assessed using a 20-item questionnaire covering humiliating, demeaning or threatening utterances in the partner relationship during pregnancy over the previous year.^29^ All participants scoring above the threshold on the measure of psychological abuse at 20 weeks’ gestation were eligible for inclusion in the intensive sample, plus a random selection from those below. Within the intensively assessed stratified subsample, 51% were drawn from the women with high psychosocial risk and 49% from those with low psychosocial risk. A variable indicating whether the mother was high or low psychosocial risk allocation to the intensive sample was included to account for the sample stratification to allow for the generalisation of results to the general population.

#### Demographic variables

Demographic variables known to be associated with maternal depression and child mental health disorders were: maternal age at first pregnancy, education (0=left education age 18 or younger, 1=left education after age 18), marital status (0=single or with partner living elsewhere, 1=married or cohabiting) and socioeconomic status assessed at recruitment at 20 weeks’ pregnancy. Socioeconomic status was determined using the revised English Index of Multiple Deprivation^28^ (IMD) and converted to quintile categories with a binary variable (1=most deprived, 0=all 4 other quintiles) used for analysis.

### Statistical analysis

Longitudinal latent class analysis (LLCA) was used to characterise maternal depression as it provides a method to identify and summarise patterns present in symptoms measured longitudinally.^20,39^ An advantage of LLCA compared with other group-based modelling techniques is that it is not based on the assumption of continuous, normal distribution. Instead, it is assumed that classes may follow different courses that vary over time and therefore allow for irregularity and change.^40^ The approach is well suited to data from prospective studies as it is based on the ‘maximum likelihood function’ that allows for data missing at random to be included.^41^ In LLCA, a larger sample size is preferable in order to increase the accuracy of hypothesised groups.^42^ Therefore, we used data from the 1233 extensive sample to model mothers’ depressive symptoms across the first year. Of the sample, 997 had reported on their depressive symptoms during the first year on at least two occasions.

As is common in community samples, maternal depression scores in this study were highly skewed. Such distributions can lead to biased model estimates and unreliable fit statistics.^39^ To address this, the use of ordinal variables to represent the actual distribution of scores has been recommended.^41^ Therefore, four ordinal categories were created to use in the LLCA based on the frequency distributions of mothers’ depression scores at each assessment point.

We performed LLCA using Mplus, Version 4.1.^43^ In LLCA, models are specified and fitted successively, with fit to the data tested against several fit indices as recommended in the literature.^39^ Models were evaluated on the basis of the Bayesian Information Criteria (BIC), entropy and the Lo–Mendell–Rubin likelihood ratio test (LMR-B). Lower BIC and higher entropy values indicate more accurate classification.^41^ A significant LMR-B indicates that the addition of a further class has made a significant improvement to the model as compared with the class solution that comes before it. After the model is chosen, participants are assigned to their most likely class according to which they receive the highest posterior probability for. A variable representing this assignment is then used to examine predictions from class membership to hypothesised outcomes.

CBCL externalising, internalising and total problems scores were skewed, and this was corrected using a log transformation. Hypothesis testing proceeded in three steps. In the first step, we examined associations between the confounders, maternal depression trajectory and child problems in multiple linear regression. Dummy variables were used that represented the mother’s most likely class membership, with the ‘very low’ class as the reference group. In the second step, the effect of adding maternal negative emotionality was examined. In the third step, the effect of adding BPD symptoms was examined. Maternal depression at 2.5 years, which may introduce reporting bias for child symptoms, was not included initially as it may also be a mediator of depression effects. However, final analyses were repeated including maternal depression scores at follow-up.

## Results

### LLCA model choice

Models with between two and six classes were estimated. Fit indices for each are presented in Table 1. Examination of the BIC, entropy and LMR-B significance suggested that the three-class model was the most adequate fit to the data. This model had the lowest BIC value, indicating a better fit to the data. Entropy of 0.72 suggested ‘medium’ classification accuracy, and the highly significant LMR-B test suggested that the three-class model was a significant improvement in fit as compared to the two-class model. The three-class model also had the highest mean posterior probabilities, further supporting this selection. The three-class model included a ‘high’ class (20.0%) made up of mothers whose posterior probability of scoring >10 on the EPDS was at least 0.5 or higher at all of the assessment points, an ‘intermediate’ class (33.4%) that had relatively low probabilities of scoring >10 on the EPDS (0–0.25) across the assessment points and a ‘very low’ class (46.6%) where mothers had consistently low probabilities (<0.1) of scoring >10 on the EPDS across all assessment points. Extraction of class membership for the 251 mothers who had provided outcome data at 2.5 years yielded 42 mothers in the high trajectory, 119 mothers in the intermediate trajectory and 90 mothers who followed the very low trajectory.

**Table 1:** Fit statistics for each model estimated using LLCA

Model	BIC	Entropy	LMR-B	LMR-B *P*
Two-class	5752.16	0.60	390.82	<0.001
Three-class	5668.10	0.72	131.07	<0.001
Four-class	5683.58	0.69	32.63	0.026
Five-class	5713.59	0.70	18.27	0.072
Six-class	5749.99	0.63	12.53	1.00

LLCA, longitudinal latent class analysis; BIC, sample size adjusted Bayesian Information Criterion; LMR-B, Lo–Mendell–Rubin likelihood ratio test.

### Descriptive statistics

The simple correlations and summary statistics for all the variables are presented in Table 2. Spearman’s Rank correlations were used throughout for continuous variables, with polychoric and tetrachoric correlations used where appropriate for ordinal and binary variables. It can be seen that maternal BPD symptoms were significantly associated with being younger at the birth of the first child, with less education, with single parent status, elevated deprivation and infant negative emotionality. Maternal BPD symptoms were also associated with membership of the high depression trajectory and maternal negative emotionality. Both maternal depression class and BPD symptoms were associated with child externalising, internalising and total problem behaviours. Maternal depression at the time of reporting was also significantly associated with the three child outcomes.

**Table 2:** Summary statistics and bivariate associations for variables used in the study^a^

	Child ext.	Child int.	Child total problems	Mother BPD symptoms	Mother depression trajectory^b^	Mother EPDS	Mother negative emo.	Child negative emo.	High risk	High deprivation	Mother age	Mother education >18 years	Married/cohabiting
Child externalising at 2.5 years	1	0.61***	0.89***	0.37***	−0.18**	0.24***	0.26***	0.26***	0.19**	0.02	−0.20**	−0.09	−0.21**
Child internalising at 2.5 years		1	0.82**	0.35***	−0.27***	0.27***	0.30***	0.21**	0.13**	0.11	−0.16**	−0.14*	−0.27***
Child total problems at 2.5 years			1	0.40***	−0.25***	0.28***	0.29***	0.27***	0.16**	0.07	−0.17**	−0.13	−0.22***
Mother BPD symptoms				1	−0.29**	0.28***	0.57***	0.19**	0.29***	0.15*	−0.23***	−0.15*	−0.25***
Mother depression trajectory					1	−0.58***	−0.52***	−0.22**	−0.18	0.09	0.08	0.05	0.28
Mother EPDS at 2.5 years						1	0.34***	0.12	−0.03	−0.05	−0.05	−0.01	−0.18**
Mother negative emotionality							1	0.30***	0.37***	0.04	−0.14*	−0.06	−0.26***
Child negative emotionality								1	0.08	0.09	−0.21**	0.01	−0.12
High-risk allocation to sample									1	0.08	−0.12	0.01	−0.23
High deprivation										1	−0.28***	−0.38***	−0.29**
Mother age											1	0.22***	0.32***
Mother education >18 years												1	0.35**
Number	251	251	251	251	251	246	243	241	251	251	251	251	251
Mean	10.31	6.05	26.5	9.96	2.19	5.19	0.76	2.82	0.42	1.62	27.94	0.47	0.77
s.d.	6.75	5.20	16.9	2.04	0.70	4.95	0.51	0.62	0.82	0.49	6.15	0.50	0.42

ext., externalising; int., internalising; BPD, borderline personality disorder; EPDS, Edinburgh Postnatal Depression Scale; emo., emotionality.

a Associations estimated using Spearman’s Rank, polychoric and tetrachoric correlations; ***<0.001; **<0.01; *<0.05.

b Mother depression trajectory coded: 1=high, 2=intermediate, 3=very low.

### Prediction of child externalising, internalising and total problem behaviours

The regression models for externalising, internalising and total child problem behaviours are shown in Table 3. In the first step, for all three outcomes and after controlling for confounders, infant negative emotionality and the high maternal depression trajectory made independent contributions to child symptoms. For each outcome, there was a modest but statistically non-significant effect of adding maternal negative emotionality to the model, and this also led to some attenuation of the contributions of infant negative emotionality and maternal high depression.

**Table 3:** Summary of multiple linear regression models predicting CBCL total, externalising and internalising problems at age 2.5 years^a^

	CBCL total problems		CBCL externalising problems		CBCL internalising problems	
	β (CI)	*P*	β (CI)	*P*	β (CI)	*P*
Step 1	ΔR^2^=0.15		ΔR^2^=0.12		ΔR^2^=0.18	
Negative emotionality – infant	0.19 (0.06 to 0.30)	0.004	0.14 (0.01 to 0.19)	0.035	0.18 (0.07 to 0.38)	0.005
High depression trajectory – mother	0.22 (0.13 to 0.58)	0.002	0.17 (0.04 to 0.37)	0.017	0.25 (0.24 to 0.80)	<0.001
Intermediate depression trajectory – mother	0.13 (−0.01 to 0.31)	0.069	0.07 (−0.06 to 0.18)	0.302	0.12 (−0.03 to 0.39)	0.089
Step 2	ΔR^2^=0.01		ΔR^2^=0.01		ΔR^2^=0.01	
Negative emotionality – infant	0.18 (0.04 to 0.29)	0.008	0.12 (−0.01 to 0.17)	0.066	0.17 (0.05 to 0.36)	0.010
High depression trajectory – mother	0.17 (0.01 to 0.52)	0.042	0.11 (−0.06 to 0.31)	0.178	0.20 (0.09 to 0.74)	0.012
Intermediate depression trajectory – mother	0.09 (−0.06 to 0.28)	0.211	0.03 (−0.10 to 0.16)	0.643	0.09 (−0.09 to 0.35)	0.238
Negative emotionality – mother	0.12 (−0.04 to 0.32)	0.134	0.13 (−0.02 to 0.24)	0.105	0.11 (−0.07 to 0.39)	0.168
Step 3	ΔR^2^=0.04		ΔR^2^=0.04		ΔR^2^=0.03	
Negative emotionality – infant	0.17 (0.05 to 0.28)	0.174	0.12 (−0.01 to 0.17)	0.064	0.17 (0.05 to 0.36)	0.010
High depression trajectory – mother	0.14 (−0.02 to 0.47)	0.074	0.09 (−0.08 to 0.29)	0.272	0.18 (0.06 to 0.69)	0.021
Intermediate depression trajectory – mother	0.11 (−0.04 to 0.29)	0.106	0.05 (−0.08 to 0.16)	0.522	0.10 (−0.07 to 0.36)	0.181
Negative emotionality – mother	−0.02 (−0.23 to 0.17)	0.790	−0.01 (−0.15 to 0.14)	0.956	−0.01 (−0.26 to 0.25)	0.964
Borderline personality disorder symptoms – mother	0.27 (0.03 to 0.12)	0.001	0.26 (0.02 to 0.08)	0.002	0.21 (0.02 to 0.13)	0.007

CBCL, Child Behavior Checklist.

a All models controlled for maternal age at first pregnancy, maternal age, education, marital status, socioeconomic status and stratification status.

With the introduction of maternal BPD symptoms in the third step, the contribution of the depression trajectory was substantially reduced for all three CBCL outcomes. In the models for externalising and total problems, with the addition of BPD symptoms the contribution of maternal depression trajectory became non-significant. BPD symptoms explained an additional 4% of variance in child externalising problems, 3% in child internalising problems and 4% in total behaviour problems. The analyses were rerun including mothers’ EPDS scores at age 2.5 years to account for possible reporting bias, and the findings were unchanged.

## Discussion

In a prospective study of parents and children recruited from the general population, an elevated trajectory of maternal depression symptoms over the first year of life, postpartum, predicted higher child externalising, internalising and total problems as reported by mothers at 2.5 years. However, maternal depression trajectory was associated with elevated BPD symptoms identified during pregnancy. This entirely explained the association between maternal depression symptoms and externalising and total problems. For internalising problems, maternal depression symptoms remained a significant predictor.

The finding in this study of an association between maternal depression and child externalising problems is consistent with evidence from a wide range of previous studies.^9–11^ Of particular relevance, Cents and colleagues,^12^ using a similar modelling approach in a general population study, showed that elevated maternal depression trajectories during infancy were associated with higher child externalising problems. There were also some differences, in that trajectories were derived from measurement over a greater time period from pregnancy up to 36 months postnatally and four trajectories were identified, three of which were associated with child symptoms. Previous studies of maternal depression, however, have not examined for the additional contribution of BPD symptoms.

### Strengths and limitations

Strengths of the study included prospective examination of maternal depression and BPD symptoms in a sample identified from the general population, and accounting for a number of plausible confounds, including infant temperament assessed as negative emotionality, and maternal negative emotionality, a measure of neuroticism. BPD symptoms were assessed in interview and rated independently of reports of child symptoms, and so were not open to effects of shared method variance, which may limit interpretation of associations between self-report measures. A limitation of the measurement of the outcome was that ratings were available only from one informant. Although this is common in studies of emotional and behavioural outcomes in young children, information from further informants may have given different findings. The study was limited also in not being able to account for shared genetic influences on BPD symptoms and child emotional and behavioural problems, and there may be further confounds for BPD symptoms that were not assessed.^44^ Participants were recruited from a geographical area with few ethnic minority families and high levels of socioeconomic deprivation, which may limit the generalisability of the findings.

### Implications

The potential importance of maternal personality dysfunction was identified more than 30 years ago in a prospective study of the children of psychiatric patients,^45^ but it has received relatively little attention since then. A recent review of studies of BPD, parenting and child adjustment found substantial evidence for reduced maternal sensitivity and increased intrusiveness associated with maternal BPD, which may contribute to increased risk for child psychopathology.^18^ However, evidence regarding links with externalising and internalising problems in early life is very limited. If the associations reported here do reflect a causal link, a major challenge will be to identify specific components of BPD that may have an impact on child adjustment. A central feature of BPD is interpersonal and emotion regulation difficulties^46^ that are likely to negatively affect the mother–child relationship and parenting behaviours.^23,47,48^ This may be particularly important in the early years, given the prominence and salience of the mother–child relationship.^49^ Associations between maternal BPD symptoms and maternal behaviours with infants such as role confusion and disoriented behaviours may be particularly relevant because of their association with disorganised attachment.^50^ Effects may also arise from associated difficulties such as relationships with partners with personality dysfunction and marital discord.^45^ A key implication for early preventative and treatment interventions is that maternal postnatal depression may be the wrong focus and that even earlier support for pregnant mothers with personality difficulties may be more relevant.
